# The Effect of the eHealth Intervention ‘MyPlan 1.0’ on Physical Activity in Adults Who Visit General Practice: A Quasi-Experimental Trial

**DOI:** 10.3390/ijerph15020228

**Published:** 2018-01-30

**Authors:** Laurent Degroote, Jolien Plaete, Ilse De Bourdeaudhuij, Maïté Verloigne, Vicky Van Stappen, An De Meester, Louise Poppe, Celien Van der Mispel, Geert Crombez

**Affiliations:** 1 Department of Movement and Sports Sciences, Ghent University, 9000 Gent, Belgium; jolien.plaete@vigez.be (J.P.); ilse.debourdeaudhuij@ugent.be (I.D.B.); maite.verloigne@ugent.be (M.V.); vivstapp.vanstappen@ugent.be (V.V.S.); a.demeester@ugent.be (A.D.M.); louise.poppe@ugent.be (L.P.); celien.vandermispel@gmail.com (C.V.d.M.); 2 Department of Experimental-Clinical and Health Psychology, Ghent University, 9000 Gent, Belgium; geert.crombez@ugent.be

**Keywords:** eHealth, physical activity, general practice, self-regulation

## Abstract

Physical inactivity is one of the major risk factors for poor health in the world. Therefore, effective interventions that promote physical activity are needed. Hence, we developed an eHealth intervention for adults, i.e., ‘MyPlan 1.0’, which includes self-regulation techniques for behaviour change. This study examined the effect of ‘MyPlan 1.0’ on physical activity (PA) levels in general practice. 615 adults (≥18 years) were recruited in 19 Flemish general practices, for the intervention group (*n* = 328) or for the wait-list control group (*n* = 183). Participants in the intervention group received the web-based intervention ‘MyPlan 1.0’ and were prompted to discuss their personal advice/action plan with their general practitioner. Participants in the wait-list control group only received general advice from the website. Self-reported physical activity was assessed with the International Physical Activity Questionnaire (IPAQ) at baseline and after one month. A three-level (general practice, adults, time) regression analysis was conducted in MLwiN. Significant intervention effects were found for total PA and moderate to vigorous PA with an increase for the intervention group compared to a decrease in the control condition. However, there was a high dropout rate in the intervention group (76%) and the wait-list control group (57%). Our self-regulation intervention was effective in increasing physical activity levels in adults. Future studies should consider strategies to prevent the large dropout from participants.

## 1. Introduction

To reduce the risk of chronic diseases and to gain health benefits, adults are recommended to accumulate at least 30 min of moderate-intensity physical activity (PA) during five days per week, or 20 min of vigorous-intensity PA during three days per week or a combination of the two [1]. However, these recommendations are not achieved by a large part of the population [2]. For example, in Flanders (Belgium), 62% of the adults do not reach these recommendations for PA [3]. Consequently, lifestyle interventions that effectively promote PA in a large number of people at low costs are required [4]. 

eHealth is the use of emerging information and communication technology, especially the internet, to improve or enable health and health care [5]. Interventions using eHealth have been found to be a promising approach to changing health behavior, including PA, on a large scale. This is because of the widespread use of the internet and related technologies that can reach a large number of people [6,7]. Notwithstanding, studies often report small effects. So, efforts should be made to increase intervention effects [6,7]. First, effects may be increased by targeting a variety of behaviour change processes, at both the pre-intentional and the post-intentional phase as defined by the Health Action Process Approach [8,9,10]. This can be achieved by adopting self-regulation techniques such as goal setting and self-monitoring [11,12]. Second, effects may be increased by implementing eHealth interventions in settings in which additional advice and support via face-to-face contact is provided [13,14,15,16]. General practice may be an appropriate setting for lifestyle interventions. General practices have a varied population, are easily accessible, have regular consultations with patients, and patients value the advice of general practitioners (GPs) [17,18,19]. However, GPs also report barriers to implementing lifestyle interventions in their practice, of which the most important is time restriction [20,21,22,23,24,25,26,27,28]. EHealth interventions may be a solution. Such interventions can take over some tasks of the GPs (such as offering advice, revising the current level of PA…), and can prompt and guide GPs to further counsel their patients [17,28,29,30,31]. 

Based upon the above reasoning, we developed the eHealth intervention ‘MyPlan 1.0’, a website to promote healthy behaviour in adults. The intervention consists of a PA module and a nutrition module, which focuses on fruit and vegetable consumption. At the start of the intervention, users have to choose which behaviour (PA, fruit consumption or vegetable consumption) they want to work on. ‘MyPlan 1.0’ is based on self-regulation theory, offering users a range of self-regulation techniques that guide them through behaviour change. ‘MyPlan 1.0’ can be disseminated in general practice via tablet computers in the waiting room, or via flyers referring to the website. The intervention was evaluated as acceptable and feasible by users, and preliminary results indicated its potential to increase PA [32]. The aim of this study is to evaluate the effect of the self-regulation-based PA module only (not the nutrition modules) of ‘MyPlan 1.0’ on PA in adults who visited their general practitioner. 

## 2. Materials and Methods

### 2.1. Study Design and Participants

To evaluate the effects of the ‘MyPlan 1.0’ intervention, a quasi-experimental trial with participants from 19 general practices was conducted between November 2014 and June 2015. The general practices were a convenience sample, recruited by using email messages, telephone calls and advertisements on association websites of GPs. They were asked to offer the ‘MyPlan 1.0’ eHealth intervention to their patients. They were asked to select suitable patients, to introduce them to the ‘MyPlan 1.0’ platform and to act as a contact point during the intervention. In total, 19 general practices, of which 6 solo practices (only one GP) and 13 group practices (more than one GP), agreed to participate in the study. Interested patients from these practices signed up by filling out an informed consent and a short socio-demographic questionnaire assessing some general information on the website. Dutch-speaking adults older than 17 years who had access to the internet were eligible. Participants were recruited by the GP, or by the researchers in the waiting room. Recruitment and baseline data collection took place between October 2014 and January 2015. Allocation occurred as follows: on one day adults were selected for the intervention group during the morning consultations, and adults for the control group during the evening consultations. The next day the order was reversed. Allocation to the intervention and control group was done using a 2:1 ratio.

The intervention group completed a questionnaire on PA and received access to ‘MyPlan1.0’. The wait-list control group completed the PA questionnaire, and electronically received general information on the benefits of being physically active and on the recommended PA levels (See Table 1). The study protocol was approved by the Ghent University Ethics Committee. Approval number of the Ghent University Ethics Committee: 670201319313). The Trial Protocol of this study is reported at ClinicalTrials.gov (“Trial Registration: ClinicalTrials.gov NCT02211040”). All participating adults filled out an informed consent. 

### 2.2. Measurements 

Sociodemographic questionnaire: The following variables were assessed: age, sex, height, weight and highest degree of education (primary or secondary education, college, university). In this study, we have used the following subdivision: primary and secondary education were recoded into low educational level and college or university were recoded into high educational level.

Physical activity questionnaire: The International Physical Activity Questionnaire (IPAQ) was used to assess total PA, vigorous-intensity PA (VPA) and moderate-to-vigorous-intensity PA (MVPA) per day [21]. The IPAQ has good reliability (intra-class range from 0.46 to 0.96) and fair-to-moderate criterion validity (Spearman rho’s ranging from 0.30 to 0.37) [33]. The total PA level per week can be calculated by multiplying the frequency (days/week) and duration (min/day) of the different activities. By summing all reported physical activities executed at moderate and vigorous intensity, the total MVPA and vigorous-intensity PA level can be obtained [34].

### 2.3. eHealth Intervention ‘MyPlan1.0’ 

‘MyPlan 1.0’ consists of three separate modules, each targeting a different behaviour: fruit consumption, vegetable consumption, and PA. ‘MyPlan 1.0’ was developed using the Health Action Process Approach model [35], which is a model that can be used to identify and categorize determinants into two phases within a self-regulations frame work: a pre-intentional, motivational phase and a post-intentional, volitional phase [10]. Therefore, users were guided in changing their behaviour via different self-regulation techniques (tailored feedback, action planning, coping planning, self-monitoring).

In the first session (T0), pre-intentional and post-intentional processes of behaviour change were addressed. Users were asked to fill out the IPAQ, after which they received feedback tailored to their level of PA. The idea was to address pre-intentional processes, to raise awareness, and to motivate adults to change their levels of PA. Post-intentional processes were addressed by formulating a personal plan (including an action plan and a coping plan). Users were requested to select one or more life domains (leisure time, transport, work, household and garden work) in which they wanted to increase their PA. 

For the personal plan, they had to formulate a specific action plan by articulating what they wanted to do (e.g., go running), when (e.g., Tuesday and Friday), where (e.g., from X to Y and back), how long (e.g., 30 min total) and with whom (e.g., alone). 

Next, to complete their personal plan, users were asked to think about possible difficult situations and hindering factors (coping plan), and were assisted in making an if-then plan (e.g., If it is raining on Wednesday evening, then I go to the gym instead of running outside).

The personal plan was emailed, and participants were offered the possibility to send the personal plan to family or friends to get social support. It was also suggested to discuss the feedback and/or personal plan with their GP. 

One week after session 1, participants were invited by email to revisit the website to complete a follow-up session (session 2, T1). During this session, feedback was provided on their accomplished change during the last week (e.g., being more or less physically active) and their goals (e.g., did or did not reach the set goal). Participants had the possibility to adjust their personal plan (action and/or coping plan) based upon the progress made, or, otherwise, the experienced difficulties during the last week. 

Session 3 (T2) was identical to session 2. It took place 1 month after finishing session 1 (or three weeks after session 2). More information on the intervention and content of the different sessions can be found in the study protocol paper [36].

### 2.4. Procedure 

Table 1 describes the study procedure. In general practice, adults received a flyer with a personal code that gave access either to the eHealth programme (intervention group), or to general information only (wait-list control group). Adults chose to log in on the website via a tablet in general practice, or via their computer when back at home or at their workplace. Adults who started on a tablet in general practice and were not able to complete the first session, could halt the programme and resume it at any time by logging in on the website. After logging in, adults chose the behaviour they wanted to change. 

Participants from the intervention group filled out the IPAQ for the first time (T0) and ran the first session of the intervention. Participants from the wait-list control group filled out the IPAQ (T0) and received general feedback on the benefits of PA and the recommended levels of PA. Participants who did not start or complete the first session after one week received a reminder email and/or telephone call. 

One week (T1) and one month (T2) after session 1, participants were invited by email to return to the website. Participants from the intervention group filled out the IPAQ again and also used the follow-up intervention, as described above. Participants from the wait-list control only filled out the PA questionnaire at T1 and T2.

### 2.5. Statistical Analyses

Kolmogorov-Smirnov tests in SPSS Statistics 22.0 (SPSS Inc., Chicago, IL, USA) revealed that the outcomes were positively skewed. Therefore, outcomes were log-transformed. Original mean values are reported in the tables for ease of interpretation. Group comparability of participants at baseline was investigated using independent sample *t*-tests (for quantitative variables) and chi-square tests (for qualitative variables). These tests were also used to conduct dropout analysis. We also checked whether the missing values randomly occurred using the Little Missing Completely At Random (MCAR). 

Finally, to investigate the effect of the ‘MyPlan 1.0’. interventions on PA levels, multilevel regression, analysis was performed (the IGLS estimation method in MLWIN version 2.32, Centre for Multilevel Modelling, University of Bristol, Bristol, UK). We used intention-to-treat analyses. In a first step, a three-level null model (general practice, adults, time) was estimated for total PA (null model 1), vigorous PA (null model 2) and MVPA (null model 3). The null models were used to show the percentage of the total variance by changes in time (level 1), differences among adults (level 2) and differences among GP practices (level 3). 

In a second step, age, gender, educational level and BMI were inserted into the models as covariates. These models (models a) were compared with their respective null model using Likelihood-ratio tests. If these tests were statistically significant, the models with covariates were considered to have a better fit than the null model. 

In a third step, time (T0, T1, T2) and condition (intervention group, wait-list control group) were included as predictors in all models (model b) to investigate whether changes in the PA outcomes (from T0 to T2) differed between the intervention and wait-list control group. This was done by exploring the interaction between time and condition (Time × Condition). To determine which models (model a–model b) had better fit, Likelihood-ratio tests were conducted. Statistical significance was set at a level of 0.05, *p*-values between 0.05 and 0.10 were considered borderline significant.

## 3. Results

### 3.1. Participants Characteristics, Response and Dropout Analysis

GPs recruited only 19 participants who started the PA intervention, of which only 8 participants completed the intervention. Furthermore, there was a selection bias. GPs seemed not to have followed the instructions to recruit every adult older than 17 years (i.e., for primary prevention). It seemed that GPs selected those patients they believed would benefit from the intervention (e.g., patients with high BMI). Therefore, it was decided not to analyze results from participants recruited by GPs. 

**Figure 1:**
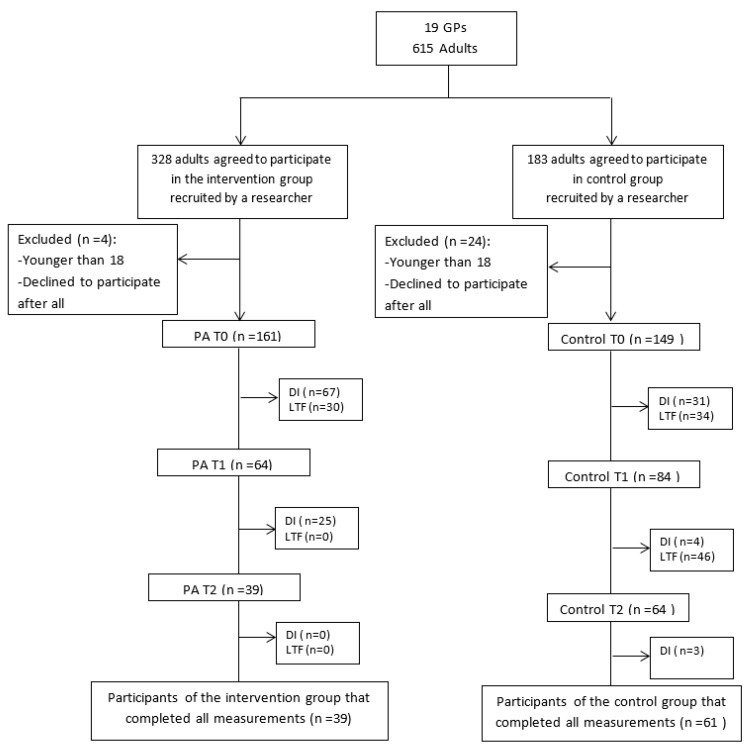
Flow of participants (DI = discontinued intervention, LTF = lost to follow-up).

The flow of all other participants is shown in Figure 1. Baseline characteristics are shown in Table 2. A little MCAR test showed that it could be assumed that missing values were completely at random [χ^2^(5) = 7.61, *p* = 0.18]. However, dropout analyses (at T2) indicated that men [χ^2^(1) = 5.04, *p* = 0.025], younger participants [t(206) = −2.64, *p* = 0.009] and participants who did not discuss their plan with their GP [χ^2^(1) = 7.90, *p* = 0.005], were more likely to drop out. No significant differences were found for the three PA outcomes, meeting PA recommendations, BMI, educational level and condition. Researchers tried to reach participants who stopped using the intervention via reminder telephone calls. The most mentioned reasons for dropout were: no time (*n* = 23), difficulties with the computer (*n* = 3), no interest (*n* = 3), lost folder with log-in code (*n* = 16), being ill (*n* = 3), forgotten (*n* = 2), too many emails (*n* = 2) and non-specific reasons (*n* = 15). 

No differences were found between the intervention and the control group at baseline, regarding age, BMI, the three PA outcomes, educational level, sex and meeting PA recommendations. 

### 3.2. Effects on PA 

The random parts of the null model showed that the variance at the time-level differed significantly from zero for total PA, vigorous PA and MVPA. Variance at the adult-level also differed significantly from zero for total PA, vigorous PA and MVPA. There was no significant between-GP variance in total PA, vigorous PA and MVPA (Table 3). 

Education was significantly related to total PA, vigorous PA and MVPA. Lower educated adults had a higher total PA level, a higher vigorous PA level and a higher MVPA level than higher educated adults (Table 3). Higher age was significantly related to lower vigorous PA levels, and lower MVPA levels (Table 3).

There was a significant interaction between time and condition for total PA and for MVPA (Table 3), indicating the change in total PA and in MVPA (from T0 to T2) significantly differed between the wait-list control group and the intervention group. Means regarding PA of both groups are displayed in Table 4. An increase in total PA and MVPA from T0 to T2 was found in the intervention group compared to a decrease in the control condition. No significant interaction effects were found for vigorous PA (Table 3), suggesting that the change in vigorous PA from T0 to T2 was not different for the intervention and wait-list control groups.

## 4. Discussion

This study investigated the effect of the self-regulation-based eHealth intervention ‘MyPlan 1.0’ on self-reported PA in adults visiting general practice. Increases in total PA and MVPA were found in the intervention group of ‘MyPlan 1.0’ compared to decreases in total PA and MVPA in the wait-list control group, which only received general information. There was no intervention effect on vigorous PA. 

The results suggest that ‘MyPlan 1.0’ was effective in increasing PA levels in adults, although there was no effect on vigorous PA. This finding may not come as a surprise, as ‘MyPlan 1.0’ does not specifically focus on increasing high intensity activities. Moreover, self-regulation theory allows individuals to set their own goals and does not impose goals that take into account the health recommendations or intensity of the activity [10]. Because users were prompted to choose a physical activity in the domains of work, active transport, leisure time or household, they are allowed to set achievable, personal goals that do not necessarily involve vigorous PA. 

To our knowledge, few eHealth interventions targeting PA have been implemented in primary care and the evidence on the effectiveness of these interventions is inconsistent [22,31,37,38,39]. The study of Glynn et al. (2014) conducted in general practices, found an increase in physical activity in adults who used a smartphone application that included automatic feedback and tracking of step counts, visual appealing graphic display of step-count history, goal setting. GPs were only involved in recruiting patients to use the smartphone application [37].

The computer-tailored programme of van Keulen et al. (2011), which was based on the Self-regulation I-change Model and Control Theory, included tailored feedback and action planning and also showed significant increases in participants’ physical activity. Those participants received a written invitation from their GP, which explained the content of the study [38]. De Cocker et al. (2012) found no effects on PA when disseminating their computer-tailored step-advice in general practice and testing this tool against no advice [39]. Parekh et al. (2012), who investigated the effect of a minimal computer-tailored intervention in a primary care setting, also did not find improvements in PA. This intervention consisted of a one-page computer-tailored feedback letter in which general guidelines, tips to reach the guidelines, information related to health benefits and links for more information, were provided [31]. Carrol et al. (2010) who tested a computer-tailored intervention, also did not find any intervention effects [22]. In that study, the intervention group received a tailored report on PA, and the control group received information on specific preventive screening tests. In the abovementioned five studies, the tailored feedback was similar to the tailored feedback provided in ‘MyPlan 1.0’. However, in ‘MyPlan 1.0’ the tailored feedback was followed by the adoption of several self-regulation techniques whereas the studies of Carroll et al. and Parekh et al. did not include any and the study of De Cocker et al., Keulen et al. and Glynn et al. only included goal setting. The implementation of self-regulation techniques in our intervention may have contributed to bridging of the well-known intention-behaviour gap [40], resulting in higher effects on PA (see also [41,42]).

Previous research, albeit not in primary care settings, has shown that interventions that include goal setting, self-monitoring, feedback, exercise prescription and cues to action, lead to more PA [12,41,42]. The effectiveness of eHealth interventions for PA may thus depend on the included self-regulation techniques. Unfortunately, the design of our study does not allow for the examination of the effectiveness of separate components or specific combinations. 

Originally, this study also wanted to investigate whether the involvement of GPs increased the effectiveness of the intervention. GPs were instructed to recruit and motivate adults to participate in the intervention, and to discuss the advice/action plan with their patients. Unfortunately, GPs did not recruit many participants. GPs only recruited 19 participants that chose the PA module, of which only 8 participants completed all sessions. Furthermore, GP’s seemed to have targeted particular individuals (e.g., individuals with a high BMI), creating a selection bias. It may well be that GPs only advertised the intervention in their general practice, and did not provide a personal explanation in regard to the study [36]. Low response rates were also reported in other studies in which computers (0–12%) [28] or flyers (6%) [15] were provided in the waiting room without personal contact. Other studies that disseminated a computer-tailored eHealth intervention in general practice and that reported a sufficient to high reach, made use of invitation letters or emails sent by GPs [22,31,39] or used multiple methods (referral by GP, self-referral in response to primary care center, community advertisements or mailshots (a dispatch of mail, especially promotional material, to a large number of people) [37]. In most of these studies [31,37,38], GPs’ tasks were limited to recruiting patients to participate in the computer-tailored study, but GPs were not further involved in the intervention itself. In our study, attempts were made to further involve GPs in the intervention by instructing participants to discuss their tailored feedback and/or personal plan with their GP [22]. Nevertheless, the frequency of discussing the advice with GPs was low (23.8%), and comparable to previous research [42]. Although GP’s were not able to recruit many patients and to provide additional advice by discussing the personal plan, it might be relevant to further investigate the possibility of implementing health promotion programmes in general practice. Research has indicated that PA counseling by GPs may make adults more open to considering changing their health behaviour [21]. The fact that an intervention is supported by the GP can increase credibility of the intervention. This might argue for a further evaluation of strategies to implement eHealth interventions in health care settings (e.g., via other health care workers such as GP assistants and nurse practitioners). 

We have to acknowledge that a high dropout rate was observed in the intervention group (76%) as well as in the wait-list control group (57%), despite the use of periodic email reminders, the use of telephone reminders, the use of incentives, and the provision of counsellor support. However, it seems that this is still not sufficient prompting, and future studies should try to incorporate more or better ways to encourage participants during the intervention. First, consistent with previous research [38,39], lack of time was the most reported reason for dropout by adults reached via telephone calls. Indeed, the research required the use of questionnaires. This may have created an extra burden for the participants as it took on average about 25 min to complete the questionnaire on demographic information, the behaviour questionnaires, to read the feedback and to make an action and coping plan. Future research needs to take into account that drop out is not necessarily due to the intervention but may simply be due to the burden of research. Therefore, the use of pedometer-based smartphone apps on mobile devices, for example, may help increase completion rates [38,39,43,44]. Another possibility is to use activity trackers, but research is needed to determine the validity and reliability to measure PA. An additional advantage of these devices is that they can be used as an intervention tool to give real-life feedback on step counts or PA level [38,39], and that they might be appealing to men and younger participants, two groups who were more likely to dropout from our study. For example, previous studies have already shown that younger people are more interested in using an activity tracker [45]. However, the fact that using these devices would reduce their dropout is just a hypothesis and should be further investigated. Second, ‘MyPlan 1.0’ was developed to bridge the intention-behaviour gap. This suggests that it could be that for adults who have no strong or even no intention to change their behaviour, the intervention is less suitable. This could possibly lead to a higher drop-out. Unfortunately, the level of intention was not measured in this study and should be included in future studies that want to bridge the gap between intention and behaviour. Similar to people having a lack of intention, the intervention may be effortful and unnecessary for those individuals who already have a habit of PA. Third, although self-regulation techniques have been shown to be effective for changing behaviour [12,46,47,48], individuals may find their use cumbersome. There is not yet a lot of research on this topic. So, future research may focus on the identification of only those behaviour change techniques that are proven to be effective in order to reduce dropout rates. Finally, other measures to avoid large dropout could be to not only focus on behaviour change theories, but also on theories that address user engagement with the intervention, for example, by applying a more person-based approach and integrating gamification elements [49].

This study has some important limitations. First, results were based on self-reported PA which can lead to response and recall biases [50]. In future research, PA can be measured via accelerometers, pedometers or validated apps. Second, post measurements were only carried out during the last session of the 4-week intervention. In future research, long term follow-up measurements are necessary. It may be that intervention effects decline or increase after intervention completion [18,51]. Third, there was no fixed number of times that the participants of the study had to visit the GP and we are also not aware if other voluntary appointments were made within the study time frame. This might be a limitation for the intervention as it could be that there was no possibility to discuss the personal plan with the GP. Further, we have no information on who filled out the questionnaires at the GP or at home, so we cannot check whether this yields other results. 

## 5. Conclusions

’MyPlan 1.0’ had positive effects on total PA and MVPA in adults. In future eHealth interventions, beliefs of GPs should be targeted and/or other implementation strategies for primary care must be evaluated, because the reach was very low when disseminating the tool in routine work-flow of general practitioners. Effective ways to prevent dropout from participants should also be considered. To develop a compact but effective intervention, it is necessary to identify (combinations of) self-regulation behaviour change techniques that are effective. 

## Figures and Tables

**Table 1:** Study Procedure.

	Intervention Group		Wait-List Control Group	
	Receive flyer with instructions to participate in the study		Receive flyer with instructions to fill out the evaluation questionnaire	
T0	Sign in on a tablet in general practice	Sign in on the website (e.g., back at home)	Sign in on a tablet in general practice	Sign in on the website (e.g., back at home)
	Selection of PA as target behavior Fill out IPAQ Receive tailored feedback Use the self-regulation tool (personal plan with action and coping plan) Receive email with personal plan		Fill out IPAQ Receive general feedback	
	Receive invitation email		Receive invitation email	
T1	Fill out IPAQ Receive tailored feedback on the behaviour change process Maintain or adapt personal plan Receive email with personal plan		Fill out IPAQ	
	Receive invitation email		Receive invitation email	
T2	Fill out IPAQ Receive tailored feedback on the behaviour change process Maintain or adapt personal plan Email with personal plan		Fill out IPAQ	

Note: IPAQ = International Physical Activity Questionnaire.

**Table 2:** Baseline characteristics of participants.

	Intervention Group	Control Group
	(*n* = 94)	(*n* = 118)
Age (mean ± SD years)	43.51 ± 14.70	46.14 ± 14.76
Gender (% male)	27.8	33.1
Education level (% high = university or college degree)	48.9	49.2
BMI (mean ± SD kg/m^2^)	25.56 ± 5.24	25.21 ± 5.10
Not meeting MVPA recommendations (%)	42.2	33.9
PA outcomes (mean ± SD min/week) Total PA	683.39 ± 500.89	733.96 ± 548.36
Vigorous PA	88.30 ± 144.93	119.13 ± 1778.66
MVPA	395.95 ± 383.63	444.41 ± 430.31

**Table 3:** Relationship with total PA, vigorous PA and MVPA.

	Total PA			Vigorous PA			MVPA		
Parameter	Null Model 1	Model 1a	Model 1b	Null Model 2	Model 2a	Model 2b	Null Model 3	Model 3a	Model 3b
**Fixed Part**	**β (S.E.)**	**β (S.E.)**	**β (S.E.)**	**β (S.E.)**	**β (S.E.)**	**β (S.E.)**	**β (S.E.)**	**β (S.E.)**	**β (S.E.)**
Intercept	706.83 (35.76)	860.42 (55.24)	903.76 (65.97)	106.80 (10.94)	136.03 (0.74)	113.42 (21.19)	418.51(35.80)	475.22 (46.56)	518.00
Age		−2.92 (2.50)	−3.04 (2.51)		−3.54 (0.74) ***	−3.65 (0.74) ***		−5.88 (1.84) **	−5.98 (1.86) **
Gender		−114.23 (77.00)	115.94 (77.11)		29.80 (22.66)	27.60 (22.60)		62.35 (55.69)	60.21 (55.78)
Education		249.38 (70.12) **	24.44 (70.10)		77.09 (20.79) **	77.20 (20.65) **		−163.50 (52.25) **	−163.54 (52.21) **
BMI		0.77 (7.10)	0.97 (7.12)		1.15 (2.10)	1.35 (2.09)		1.92 (5.26)	2.19 (5.28)
Condition			−78.91 (75.48)			42.22 (22.74) *			−64.62 (59.78)
Time			−62.14 (33.29)			12.22 (12.87)			−57.10 (24.31) **
Time * group			103.38 (50.73) **			24.07 (17.06)			74.97 (37.04) **
**Random Part**	**σ2 (S.E.)**	**σ2 (S.E.)**	**σ2 (S.E.)**	**σ2 (S.E.)**	**σ2 (S.E.)**	**σ2 (S.E.)**	**σ2 (S.E.)**	**σ2 (S.E.)**	**σ2 (S.E.)**
Time-level variance	68,746.27 (6741.13) ***	65,198.83 (6487.53) ***	63,733.49 (6341.72) ***	7963.66 (780.901) ***	7282.02 (724.59) ***	7209.91 (717.41) ***	36,057.49 (3384.52) ***	34,889.68 (3326.60) ***	33,968.72 (3238.79) ***
GP-level variance	0.000 (0.000)	0.000 (0.000)	0.000 (0.000)	0.000 (0.000)	251.51 (723.52)	166.69 (689.42)	11,231.87 (7735.48)	9516.69 (7009.75)	8949.41 (6832.96)
Adult-level variance	231,589.33 (26,296.67 ) ***	212,810.52 (24,633.71) ***	213,365.22 (24,606.65) ***	20,896.51 (2471.57) ***	17,457.61 (2224.60) ***	17,357.36 (2209.51) ***	127,582.45 (3384.52) ***	118,342.31 (13,611.74) ***	118,918.18 (13,614.77) ***
Deviance test model	6239.62	6032.69	6027.95	5298.45	5096.50	5092.459	6538.46	6313.52	6307.321
χ^2^ (df)		206.93 (4) ***	211.67 (7) ***		201.95 (4) ***	205.99 (7) ***		224.94 (4) ***	231.14 (7) ***

* *p* = 0.05–0.10; ** *p* ≤ 0.05; *** *p* ≤ 0.001.

**Table 4:** Mean PA levels in intervention and control group.

		Intervention Group (S.E.)	Wait-List Control Group (S.E.)
Total PA (min/week)	Pre	683.39 (57.70)	733.95 (50.39)
	Post	731.43 (57.70)	678.82 (50.39)
Vigorous PA (min/week)	Pre	404.79 (47.59)	445.10 (42.38)
	Post	426.64 (47.59)	391.42 (42.38)
MVPA (min/week)	Pre	88.30 (17.87)	119.13 (15.61)
	Post	105.98 (17.87)	109.21 (15.61)

## Availability of Data and Materials

The dataset(s) supporting the conclusions of this article is (are) included within the article (and its additional file(s)).

## Abbreviations

IPAQ	International Physical Activity Questionnaire
PA	physical activity
VPA	vigorous physical activity
MVPA	moderate to vigorous physical activity
GP	general practitioner
